# Assessment of the Living Conditions in Polish and German Transborder Regions in the Context of Strengthening Territorial Cohesion in the European Union: Competitiveness or Complementation?

**DOI:** 10.1007/s11205-022-02889-7

**Published:** 2022-02-10

**Authors:** Marta Gwiaździńska-Goraj, Aleksandra Jezierska-Thöle, Małgorzata Dudzińska

**Affiliations:** 1 Institute of Spatial Management and Geography, University of Warmia and Mazury in Olsztyn, Prawocheńskiego 15, 10-720 Olsztyn, Poland; 2 Institute of Geography, Kazimierz Wielki University, Plac Kościeleckich 8, 85-033 Bydgoszcz, Poland; 3 Institute of Spatial Management and Geography, University of Warmia and Mazury in Olsztyn, Prawocheńskiego 15, 10-720 Olsztyn, Poland

**Keywords:** Spatial policy, Territorial cohesion, Material living conditions of the population, No-material living conditions of the population, Evaluation, Border regions, Poland, Germany, Socio-economic geography

## Abstract

The European Union's regional policy aims to strengthen economic, social, and territorial cohesion and equal space development opportunities. It is an action linking UE that emphasises the problem of cohesion in the interregional context. The essence of territorial cohesion is the necessity to eliminate inequalities between the living conditions of the population. The concept of quality of life is ambiguous, multidimensional and interdisciplinary. This problem is of particular importance concerning border areas, i.e. the periphery of countries, which often adversely affects the population's standard of living. The article aimed to assess the living conditions and the direction of changes in those terms on the Polish-German border, an internal border of the EU (former Eastern Bloc countries). The analysis covered the years 2004–2019. The impact of the EU's regional development and cohesion policies for border areas (INTERREG) in improving the analysed regions' living conditions was also determined. The method of aggregation, standardised sums, was used in the analysis. The research showed that the level of living conditions of these border regions' population was spatially diversified, being more favourable on the German side. The analysed border regions had less favourable material and non-material living conditions than the countries' average value. Significant changes in the living conditions of border regions in the years 2004–2019 were established towards equalising the quality of life, which was influenced by the spatial policy of territorial cohesion.

**Supplementary Information:**

The online version contains supplementary material available at 10.1007/s11205-022-02889-7.

## Introduction

One of the essential elements of the European Union's regional policy is strengthening economic and social cohesion and equalising development opportunities in space (Single European Act, 1987). In 2007, the Treaty of Lisbon (2007) and the Europe 2020 Strategy (Europe) introduced the third dimension, the so-called territorial cohesion, treated as a concept of integrated development policy. Its purpose is ensuring territorial units' development and meeting their inhabitants' needs. According to European Commission (2008), territorial cohesion means "ensuring the harmonious development of all these places and ensuring that their inhabitants will be able to make the best use of the characteristics of these areas". According to the Green Paper (EC, 2008), territorial cohesion consists of equalising the population's standard of socio-economic life, integrating and shaping justice in the territorial system. On the one hand, it is related to the economic dimension of development planning and management and, on the other hand, to the concept of an integrated approach to development (Greta & Tomczak-Wozniak, 2014) In the EU context, it is indicated that the category of territorial cohesion does not mean a simple equalisation of social and economic differences in space, but the coherent development of UE as one organism (megaregion) (Raczyk et al., 2012). A new element of the future EU regional policy is the action linking Europe, which emphasises cohesion in the interregional context. The emphasis is on the integrated development of areas as spaces in which citizens of different nationalities live. In this context, new forms of cooperation between states are indicated to reduce disparities between regions (Kurowska-Pysz et al., 2017; Medeiros, 2016). The socioeconomic perspective on peripherality focuses on the dynamics of unequal socioeconomic development between core/non-core regions (Popescu et al., 2021). The essence of territorial cohesion is the necessity to eliminate inequalities between the living conditions of the population. This process in the EU countries is stimulated by various financial, organisational, and legal instruments (Kelemen, 2011) Endogenous (geographic and natural) and exogenous (socio-economic and financial) conditions mean that individual areas show different socio-economic development and quality and living conditions. The effect of the uneven development of living conditions in territorial systems is polarisation and disproportion, leading to depopulation and social, economic and natural degradation (Gwiazdzinska-Goraj et al., 2020). To eliminate these processes, the EU implements a cohesion policy, the instruments of which are to counteract the growth of differences and reduce disproportions, e.g. in the quality and living conditions of the population.

The assessment of the population's quality and living conditions was the subject of numerous scientific studies of the twentieth century (Andrews & Withey, 2005; Cambel et al., 1976; Lepper, 1998; Levy & Guttman, 1975). These studies were undertaken by economists (Ostasiewicz 2002, 2013; Piasny, 1993;), geographers (Jezierska-Thöle, 2018), sociologists (Borys, 2008; Barcaccia et al., 2013; Dziurowicz-Kozłowska, 2002; Petelewicz & Drabowicz, 2016; medycy (Schipper, 1990) and philosophers (El-Osta et al., 2007; Goetzke & Islam, 2017). Based on social sciences, the quality of life was associated with lifestyle, sense of well-being or satisfaction with the conditions and the possibility of satisfying the essential life needs (Gilbert, 2008). In medical sciences, the analysis concerned the subjective sense of healthy people's quality of life and those with chronic, incurable diseases (Health-Related Quality of Life—HRQoL) (Nordenfelt, 1993). From the economic and geographical point of view, the concept of quality of life was understood primarily in terms of quantity, and was used in economic and planning studies. The research results were necessary to conduct policies supporting the development of these areas in the field of, among other things, new concepts of spatial development in post-socialist countries in the pre-accession period (Gans & Schmitz-Veltin, 2006; Rosner, 2002). Research on the population's quality of life proved to help develop programs for reducing development differences in the new federal states (former East Germany) after Germany's reunification in 1990. Similarly, in Poland, achieving economic and social cohesion with EU countries required thorough research on development differences, including quality of life. Research on the population's quality of life covering EU member states was presented in Chmielewska (2013), Kozera and Kozera (2014), Górska (2018) and border areas by Janusz (2015), Kusterka-Jefmańska and Jefmański (2014), Goetzke and Rave (2013), Jakubowski and Bronisz (2015), Jakubowski and Miszczuk (2016), Pomianek (2019).

Determining the assessment of the quality of life was based on the UN definition of 1954. It reads as follows: "The concept of the standard of living covers the entirety of the real living conditions of people and the degree of material and cultural satisfaction of their needs through a stream of paid goods and services, as well as from social funds". Similarly, Luszniewicz (1982) defined the standard of living as the degree of meeting the material and cultural needs of households and distinguished seven basic types of needs: food, safety, health protection, housing, communication and transport, education and culture, and the environment. Similarly and Piasny (1993) indicated that "quality of life" is a complex concept conditioned by several social and economic characteristics. Ostasiewicz (2004) defines the quality of life as the quality of everything that defines human life and the quantity of everything needed for life. Through The Europe 2020 Strategy (The Europe 2020 Strategy), the EU formulates three additional goals: “intelligent growth, sustainable growth, and inclusive growth”. The strategy emphasises the social dimension in Europe and strives for a balance between the economic, labour market policy, and social aspects, and places particular emphasis on the areas of “financial poverty” and "living conditions,”labour market access,” and “education”. Accepting the population's level of living conditions as the social function as the goal of socio-economic development is, in fact, synonymous with treating development as the primary determinant of the quality of life (Borthwick-Duffy, 1992). Socio-economic development affects the change of living conditions directly by providing income to the population. It also indirectly does it by stimulating and activating economic activity, social and technical infrastructure, and improving or deteriorating the natural environment's condition. The above considerations prompted the authors to discuss the living conditions of Poland's border regions with Germany and demonstrate the importance of the EU spatial cohesion policy of cross-border areas, which aims to activate the unused socio-economic potential and strengthen the process of cooperation towards harmonious development. The region located near the border of states, particularly the cross-border zone, is in the competitive and complementary relationship within the cooperation framework, accompanied by changes in the population's living conditions. Factors that played a significant role in the selection of the research area included location conditions (internal border of the European Union) and historical factors (common history, in which a unique role was played by mass migrations after World War II and belonging to the bloc of socialist countries—East Germany and Poland).

Concerning the adopted research area, the core and periphery theory developed by Prebisch is essential for understanding the processes shaping the population's living conditions. This theory was based on the existing economic relationship between the centre and the hinterland to exploit labour resources and raw materials. Later, this theory was developed by Friedman, who, apart from the economic factor, also considered other aspects influencing the formation of the periphery's dependence on the centre, e.g. sociological, psychological and political (Grosse, 2007; Pastuszka, 2009). As a result, the periphery's development depends on the central areas in various spheres, including economic, cultural, and political, which prevents their proper development and translates into the population's living conditions (Medeiros, 2016). Simultaneously, if such a differentiation persists for too long, it contributes to the deepening of regional disproportions, reflected in Perroux's theory of polarisation, further developed by Hirschman. The principle of cumulative causality plays a unique role in the processes and phenomena in border regions, which can undoubtedly be classified as peripheral concerning a given country's economic centres. Myrdal, in his theory, points out that a change in one quantity causes a change in another quantity. However, they take place in the same direction, so they are based on feedback. Thus, positive changes accumulate in the growth process, and adverse changes accumulate in the recession (Churski, 2005). According to Myrdal, the higher the level of polarisation recorded in an area, the more difficult it is to stop the vicious circle mechanisms. Therefore, an important instrument to stop the negative processes taking place in peripheral areas, including border regions, is shaping the spatial policy; the critical element is to understand regional competitiveness and support the processes of reducing development differences. Territorial competitiveness is a process taking place in space (Bristow, 2005; Miszczuk & Jakubowski, 2019; Porter, 2003). Moreover, it denotes the economy and society's ability to increase the population's standard of living (Malecki, 2000). In international systems, competitiveness means the ability of states to produce and distribute goods and provide services in the international economy in competition with goods produced in other countries, in a way that ensures a growing standard of living (Malecki, 2000). The location is a source of many limitations and opportunities that modify development's socio-economic conditions concerning border regions. Border regions reflect the impacts of state borders on political spaces, economic relationships, and social life (Prokkola, 2019).The existence of the border reduces the economic efficiency of these regions. Borderlands constitute an area of contact between territorial units operating in potentially very different conditions shaping competitiveness. On the one hand, the dissimilarity of competitive conditions may constitute an element facilitating the establishment of cooperation relations even in a situation of increased mutual competition and, consequently, the emergence of conditions for creating a cross-border region (economic entities may use different economic conditions, e.g. lower labour costs). On the other hand, it may also result in a lack of interest in building good neighbourly relations and strengthening the functioning of units independent of each other. The result is an unnecessary and unjustified imitation of the duplication of public facilities. Hence the answer to the question in the paper title whether we deal with competitiveness or supplementation in border regions is crucial for shaping the spatial policy of territorial cohesion.

The research's main objective was to assess the population's living conditions in a dynamic perspective in the spatial policy of territorial cohesion aimed at cross-border areas. Funds and programs for EU member states are an essential implementation instrument. The following research questions were asked in the paper:

- Are there differences in the living conditions of the population of border regions related to the accession to the EU by Germany (former East Germany in 1990) and Poland (2004)?
- In what direction are the living conditions of the population changing? What are their dynamics in the spatial arrangement?
- What role and contribution do EU funds (INTERREG) play in cross-border cooperation programs to improve the population's living conditions in border regions?
- Are the changes in the shaping of Poland and Germany's border regions improve the population's living conditions, and do they result from a spatial policy based on competitiveness or supplementation?

The research problem related to the identification of factors influencing the changes in the living conditions of the border regions of Poland and Germany also applies to other border regions within the EU, which cover 40% of the EU territory and are the place of residence of 30% of the EU population—i.e. 150 million people (EC, 2008). The implementation of spatial policy assumptions, the implementation instrument of EU programs and funds, has a significant impact on shaping the population's living conditions. The research results may help planners and representatives of regional self-governments implement EU social policy, including cross-border cooperation. The research problem undertaken in the article extends the scope of research conducted by the authors in the border regions of Poland (Gwiaździńska-Goraj et al., 2020; Pawlewicz et al., 2020; Thöle & Jezierska-Thöle., 2019).

## Study Area and Methods

### Study Area

**Fig. 1:**
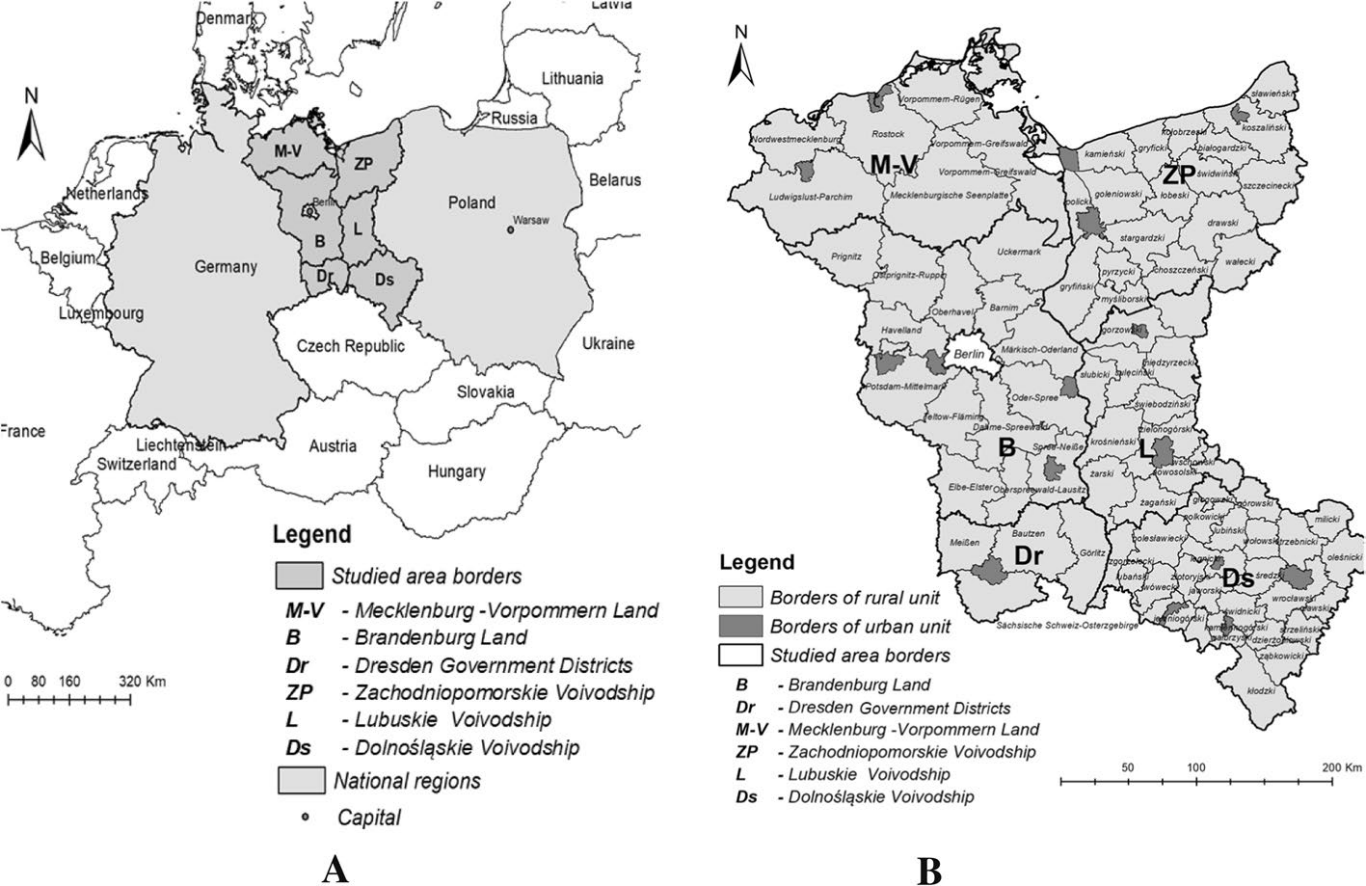
**a** Level NUTS 0 and NUTS 2; **b** Level LAU. Studied area. Source: Own elaboration

The study area is located in the European Union along Polish-German borders and covered (NUTS 2 regional level):

- *Federal states* Brandenburg, Mecklenburg-Vorpommern (Germany),
- *Government districts* Dresden (Germany),
- *Voivodship* Zachodniopomorskie, Lubuskie, Dolnośląskie (Poland) (Fig. 1a).

The regions (federal states and government districts) on the German side of the border have an area of 71 397.1 km^2^, are inhabited by about 9.9% of the German population, and their population density is 115 persons per km^2^ (the German average is 233 persons). The rate of urbanisation is 74.3% (the German average is 58.7%). The regions (voivodeships) on the Polish side of the border have an area of 56 840 km^2^, are inhabited by about 14% of the Polish population, and their population density is 99 persons per km^2^ (the Polish average is 123 persons). The rate of urbanisation is 68% (the Polish average is 60%).

The study area is located in the European Union along Polish-German borders, and it belongs to Euroregions which are trans-border associations that cooperate in the contiguous territories of European countries. The analyzed border areas belong to four Euroregions: Pomerania; Pro Europa Viadrina; Sprewa-Nysa-Bóbr; Nysa.

The analyses conducted in the selected trans-border regions at NUTS 2 and LAU (until 2016 in Poland and Germany this data aggregation level was known as NUTS level 4). LAU levels covering 80 local rural administrative units (Germany—24, Poland—56) were presented in a tabular form and in spatial distribution (Fig. 1b). Urban units at LAU were not included in the analysis.

### Methods

**Fig. 2:**
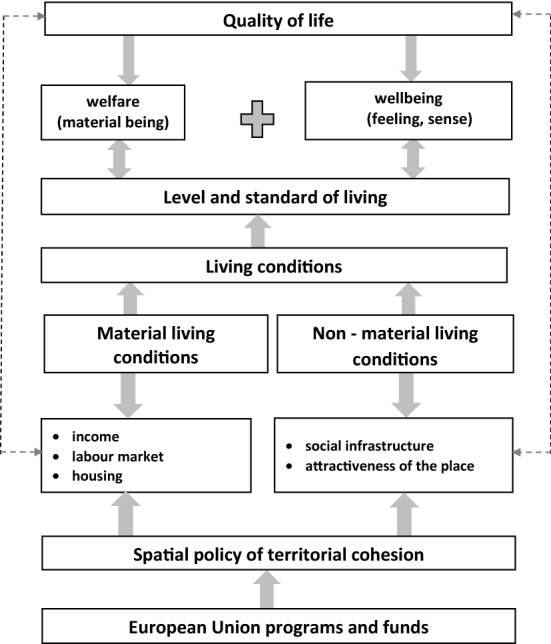
Research procedure and definition of the living conditions. Source: Own elaboration

In the literature, various definitions of life quality depend on the aspect adopted and may include both, jointly or separately, welfare issues—an analysis of an objective nature, and as wellbeing—an analysis of a subjective nature. Based on the extensive literature on the subject (Brambert & Kiniorska, 2018; Buettner & Ebertz, 2009; Hu & Wang, 2020; Jongudomkarn & Camfield, 2006; Murgaš & Klobučník, 2016; Petelewicz & Drabowicz, 2016), it can be concluded that defining and the methods aimed at assessing the quality of life may be different, depending on the research problem undertaken. Therefore, an essential element of the work was to illustrate the relationship between the quality of life and living conditions, which were determined by material and non-material conditions, taking into account the impact of the EU's spatial policy of territorial cohesion, an essential instrument of which are programs and funds of the European Union (Fig. 2).

The research was carried out in the following stages:

#### Diagnosis of the Living Conditions of the Population in the Border Regions of Poland and Germany

### Identification of Diagnostic Indicators to Assess the Living Conditions of the Population of Border Regions on the Polish and German side

The assessment of the living conditions of the border regions' population was presented in two aspects: material and non-material. The issues relating to income, labour market and housing conditions were adopted to assess material conditions. The issues related to the accessibility of social infrastructure and its attractiveness were adopted to assess the non-material conditions. Diagnostic indicators were selected to objectively reflect the population's living conditions, which was limited by the availability of data at LAU in Poland and Germany's national statistical systems (Table 1). The indicators were based on Poland's statistical data from the Local Data Bank of the Central Statistical Office (GUS) (https://bdl.stat.gov.pl/) and on Germany statistical data from The Federal Statistical Office and (https://www.destatis.de/). The statistical data in the analysed countries needed to be defined identically. The research's time scope covered 2004 (baseline—Poland's accession to the European Union) and 2019 (present state). This research approach allowed for the identification and characterisation of the dynamics of changes taking place in space. Then, quasi-constant variables were eliminated, i.e. those that do not bring new information about the studied phenomenon to the analysis. For this purpose, the coefficient of variation was calculated for each indicator while eliminating the features for which the coefficient value did not exceed 0.1. The strength of the relationship between the remaining variables was tested using the Pearson correlation coefficient. The value | 0.6 | was assumed as critical.

**Table 1:** Diagnostic indicators for the assessment of the living conditions of the population of border regions of Poland and Germany. *Source*: owne study based on: Churski (2005), Mitrică et al. (2020), Buettner and Ebertz (2009, Gwiaździńska-Goraj and Jezierska-Thöle (2013), Jezierska-Thöle (2011)

Topic	Diagnostic indicators	Character of indicators	Information on internet statistical databases for Germany, and Poland	Choice of indicators
1	2		3	4
*Material living conditions*				
Income	Average monthly gross wages and salaries	Stimulant	Unavailable	Rejected
	Income per capita	Stimulant	Unavailable	Rejected
	Vehicles per 1 000 inhabitants	Stimulant	Available	Accepted
Labour market	Registered unemployment rate	Destimulant	Available	Accepted
	Employed persons per 1000 person	Stimulant	Unavailable	Rejected
	Entities for 10 thousand population at working age	Stimulant	Unavailable	Rejected
	The share entities newly registered in total entities (%)	Stimulant	unavailable	rejected
Housing	Number of rooms per person	Stimulant	Available	Accepted
	Dwellings fitted with bathroom—in % of the total number of dwellings	Stimulant	Available	Rejected
	Dwellings fitted with central heating—in % of the total number of dwellings	Stimulant	Available	Rejected
*Non—material living conditions*				
Social infrastructure	Population per 1 hospital bed	Destimulant	Available	Accepted
	Population per 1 doctor entitled to practise medical profession	Destimulant	Available	Rejected
	Number of primary schools per 100 km^2^	Stimulant	Available	Accepted
	Number of pupils per 1 primary school unit	Destimulant	Available	Rejected
	Number of kindergartens per 100 km^2^	Stimulant	Available	Rejected
	Activities of centres of culture, cultural centres and establishments, clubs and community centres per 100 km^2^	Stimulant	Available	Rejected
Attractiveness of the place	The share of forests in the total area (%)	Stimulant	Available	Accepted
	The share of legal protected area in total area (%)	Stimulant	Unavailable	Rejected
	Population using sewage treatment plants in % of the total population	Stimulant	Available	Accepted
	Gas emissions in tonnes per year from plants of significant nuisance to air quality	Destimulant	Unavailable	Rejected
	Dust emissions in tonnes per year from plants of significant nuisance to air quality	Destimulant	Unavailable	Rejected
	Fatalities in car accidents per 100 thousand population	Destimulant	Available	Accepted
	Net migration per 1000 population	Stimulant	Available	Accepted
	Migration efficiency coefficient	Stimulant	Available	Rejected

Synthetic statistics of data by year are available in the Appendix (LAU areas).

### Comparative analysis of the Living Conditions of the Population Based on the Diagnostic Indicators at NUTS 0 and NUTS 2

The diagnostic indicators adopted in the study were used to assess the living conditions of the population based on their components: material and non-material conditions (Table 1). Because border regions often show unfavourable living conditions compared to other regions of the country, the level of border regions (NUTS 2) was compared to Poland and Germany's average values (NUTS 0).

### Delimitation of the Living Conditions of the Population in the Spatial Aspect at LAU

Based on the statistical procedure, some of the indicators adopted for assessing the population's living conditions were eliminated. As a result, a set of nine indicators was obtained. Two groups of issues were adopted to construct synthetic component indices to delimit the state of the living conditions of the population:

- *Material conditions (w1)* including diagnostic indicators: income, labour market, housing conditions: x_1_—vehicles per 1000 inhabitants; x_2_—registered unemployment rate; x_3_—number of rooms per person;
- *Non-material conditions (w2)* taking into account diagnostic indicators: social infrastructure and attractiveness of the place: x_4_—population per 1 hospital bed; x_5_—number of primary schools per 100 km^2^; x_6_—the share of forests in the total area (%); x_7_—population using sewage treatment plants in % of the total population; x_8_—fatalities in car accidents per 100 thousand population; x_9_—net migration per 1000 population.

The diagnostic indicators included both stimulants and destimulants. Diagnostic indicators were expressed in different units. Therefore these indicators were normalised and adjusted for comparability by removing the appropriate measurement units and standardising all variables by transforming them into stimulants. It was done using the zero unitarisation method, where the following transformation operations were applied:

For stimulants:

1 $$v_{ij} = \frac{{x_{ij} - \min x_{ij} }}{{\max x_{ij} - \min x_{ij} }}.$$

For destimulants:

2 $$v_{ij} = \frac{{\max x_{ij} - x_{ij} }}{{\max x_{ij} - \min x_{ij} }}.$$

where

*v*_*ij—*_standardized value of the indicator x_ij,_

*x*_*ij—*_value of the jth diagnostic indicator of an ith object.

min*x*_*ij*_*—*minimum value of the jth diagnostic indicator x_ij,_

max_*ij*_*—*maximum value of the jth diagnostic indicator xij,

The partial indices were used to calculate the synthetic indicators (w1, w2). The literature on synthetic indicators offers a wide range of aggregation methods (Wu & Barnes, 2011). The standardised sum method (Perkal indicator) was used according to the following formula.

$$w = \frac{{\mathop \sum \nolimits_{j = n}^{n} V_{ij} }}{n}$$

w—synthetic component indicators (w1 or w2).

vij—standardised value of indicators in the ith case and the jth variable;

n—number of features included in the analysis.

As a result, the above diagnostic indicators within the adopted issues were transformed into two synthetic component indicators w1, w2, respectively. One general synthetic indicator of the population's living conditions was constructed (w3), covering all diagnostic indicators (x_1_–x_9_). For this purpose, the above-mentioned statistical procedure was adapted.

Next, the areas were classified according to the living conditions of the population. Four classes (I–IV) of LAU areas were defined so that the area in one class had a similar level, using three medians (Table 2). The set of objects contains two subsets: the first is objects to which measures higher than the overall median correspond, and the second comprises all other objects. Next, intermediate medians were defined for each of the groups. Class I meant units with the lowest level, and class VI represented units with the highest living conditions.

**Table 2:** Groups of types. *Source*: based on: Młodak (2006)

Class	Classification criterion	Evaluation
I	${\text{w}}_{i} \le med_{2} \left( {w_{i} } \right)$	Unfavourable
II	$med_{2} \left( {w_{i} } \right) < {\text{w}}_{i} \le {\text{med}}\left( {w_{i} } \right)$	Medium low
III	${\text{med}}\left( {w_{i} } \right) < {\text{w}}_{i} \le med_{1} \left( {w_{i} } \right)$	Medium high
IV	${\text{w}}_{i} \ge med_{1} \left( {w_{i} } \right)$	High

$med_{1} \left( {w_{i} } \right), med_{2} \left( {w_{i} } \right)$—intermediate medians of synthetic indices

${\text{med}}\left( {w_{i} } \right)$—median of synthetic indices

#### Assessment of the Impact of the Spatial Policy of Territorial Cohesion—European Territorial Cooperation

The importance of cohesion policy was assessed based on the impact of one of its instruments, the INTERREG (European Territorial Cooperation) program. The evaluation concerned the absorption of funds by the border regions of Poland and Germany. The differentiation of the features of the areas at LAU (different area, structure, and number of inhabitants) confirms the legitimacy of selecting the relative indicator to determine the level of support the analysed areas get from cross-border programs. Therefore, the analysis specifies the support per inhabitant from LAU area.

#### Identification of the Relationship Between the Living Conditions of the Population and the Spatial Policy of Territorial Cohesion—European Territorial Cooperation

The relationship between the living conditions of the population and the spatial policy pursued within border regions, an essential instrument of which are EU funds and programs, was examined based on the relationship between the synthetic indicator of the living conditions of the population (w3) and the relative value of the relativised indicator illustrating the amount of funds provided to shape cross-border cooperation in border regions.

## Results and Discussion

### Diagnosis of the Living Conditions of the Population of Border Regions in Poland and Germany

It was examined whether the peripheral location of border regions is reflected in the population's living conditions. The level of indicators of border regions (NUTS 2) was compared to the average sizes of Poland and Germany (NUTS 0), and the spatial differentiation of the living conditions of the border area population was visualised at LAU, broken down by material and non-material conditions.

#### NUTS 2 and NUTS 0

The first group of issues is material conditions (Table 3) relating to the population's wealth in border regions. Indicators for border regions' values did not differ significantly from Poland and Germany's average, both in 2004 and 2019. Concerning the indicator of vehicles per 1000 population, border regions' situation was more favourable than across the country, which could have been caused by the need for transportation accessibility due to, for instance, people commuting to work from Poland to Germany on a daily basis. After Poland’s EU accession, the employment market in Germany opened to Polish frontier workers, which increased cross-border commuter traffic (Franke et al., 2020). In addition, a high supply and accessibility of used vehicles from Germany fostered an increase in the number of new vehicles. In the case of the unemployment rate or the number of rooms per person, the situation was less favourable than the national average. Among the border regions on the Polish side, Zachodniopomorskie, which was burdened with the State Farms (PGR) in the past, showed unfavourable material indicators, which influenced the level of wealth of the region's population. Simultaneously, there was a significant improvement in the unemployment rate and the number of rooms per person in 2004–2019, compared to the number of vehicles per 1000 population. A decline in the level of unemployment in Polish border regions was related to the economic revival and an increased rate of migration after EU markets opened for Poles. In addition, new jobs could be financed by structural funds. Similarly, in the eastern lands of Germany the release of millions of workers on the employment market contributed in a short time to the emergence of a new phenomenon called unemployment in transition by economists (Becker, 2000).On the German side, the disproportion in terms of material indicators between regions is not highly significant. However, Brandenburg and Mecklenburg had a less favourable unemployment rate compared to Dresden (Table 3). Despite the developed welfare state, access to the employment market still varies strongly in different regions. It is alarming that after 30 years since the integration of Germany, the rate of unemployment in German border regions (9.0%) is higher than in Germany (6.4%). Spatial disparities in the structure of unemployment are due to the wide-scale restructuring of state-owned and cooperative farms of the former GDR (German Democratic Republic).

**Table 3:** Indicators assessing material living conditions in border regions at NUTS 2 in Poland and Germany. *Source*: based on: www.stat.gov.pl, www.destatis.de

Specification	Diagnostic indicators—material living conditions					
	Income		Labour market		Housing	
	Vehicles per 1000 inhabitants		Registered unemployment rate		Number of rooms per person	
	2009	2019	2004	2019	2004	2019
Poland	432.2	634.7	19.0	5.2	1.2	1.5
Dolnośląskie	433.3	655.0	22.4	4.6	1.2	1.6
Lubuskie	444.2	678.9	25.6	4.9	1.2	1.5
Zachodniopomorskie	400.7	602.8	27.5	6.8	1.2	1.5
Germany	505.1	689.0	10.5	6.4	2.1	2.5
Branderburgia	515.9	586.0	18.7	9.0	2.0	2.4
Meklemburgia	485.6	559.1	20.5	10.1	2.0	2.0
Dresden	483.0	578.3	19.4	7.8	2.1	2.3

**Table 4:** Diagnostic indicators for assessing the living conditions of the population in terms of non-material conditions in border regions at the NUTS -2 level, as well as Poland and Germany. *Source*: based on: www.stat.gov.pl, www.destatis.de

Specification	Diagnostic indicators—non-material living conditions											
	Social infrastructure				Attractiveness of the place							
	Population per 1 hospital bed		Number of primary schools per 100 km^2^		The share of forests in the total area (%)		Population using sewage treatment plants in % of the total population		Fatalities in car accidents per 1000 population		Net migration per 1000 population	
	2005	2019	2009	2019	2004	2019	2004	2019	2011	2018	2004	2018
Poland	212.6	207.5	4.7	4.6	28.7	29.6	59.0	74.5	10.9	7.5	− 0.3	0.1
Dolnośląskie	204.2	198.3	4.4	4.4	29.1	29.8	73.9	81.9	8.9	7.0	− 0.5	1.3
Lubuskie	233.1	231.0	2.6	2.6	48.7	49.3	62.7	77.2	11.9	7.8	− 0.4	− 1.0
Zachodniopomorskie	216.3	216.0	2.4	2.4	34.7	35.7	59.4	83.0	9.9	7.6	− 0.8	− 0.6
Germany	157.4	166.6	4.7	4.3	29.8	30.6	96.5	96.5	5.0	3.9	1.0	4.8
Branderburgia	165.9	163.5	1.6	1.7	35.1	35.5	82.5	88.2	8.6	5.7	0.3	8.4
Meklemburgia	166.8	156.5	1.6	1.4	21.4	21.9	83.7	89.1	8.9	5.3	− 5.0	4.8
Dresden	164.7	170.8	6.2	7.5	27.9	30.9	84.7	91.7	3.9	4.8	− 5.2	3.8

The second group of issues are non-material conditions, including social infrastructure and the place's attractiveness (Table 4). The availability of social infrastructure in 2004 and 2019 was lower than the national average for both border regions. The number of patients per hospital bed was lower in the German border regions, while the number of primary schools per 100 km^2^ was higher in the Polish regions. Ensuring sufficient health care is essential due to the increasing process of “aging of society”. A decline in the number of schools is observed mostly in depopulated areas. The decreasing accessibility of basic social infrastructure simultaneously leads to an outflow of people of reproductive age and increases migration. It should be emphasized that the number of schools and educational facilities declined in the first decade of the political system transformation in the former GDR as a result of the outflow of people (mainly young people) to West Germany and big cities (Jezierska-Thöle & Janzen, 2012). The attractiveness of the place is more and more often perceived through the prism of environmental and security conditions, and the migration balance is an important indicator reflecting it. Among the adopted diagnostic indicators illustrating the place's attractiveness, no clear difference was noted between the border regions and the national average values. When comparing Polish and German border areas, significant disproportions were noted, with more favourable values in German regions. This disproportion concerned mainly the migration balance per 1000 population. In 2004, negative net migration was recorded in Polish regions. This state of affairs may be because Poland acceded to the European Union, and Polish citizens gained an opportunity to work in other EU countries, which contributed to the outflow of people from border regions. Negative values of the net migration in 2004 were obtained for Mecklenburg and Dresden on the German side. Only in Brandenburg, a positive net migration rate was recorded. Since it is a region of 'close influence' to the capital city of Berlin, it has incomparably more significant development opportunities in terms of infrastructure, and social and economic spheres, which strongly influences the demographic changes of the region. On the Polish side, no significant changes in the migration rate were recorded, except for Dolnośląskie. In German regions, a dramatic improvement was noted. In Brandenburg, this indicator was positive (as much as 8.4). On the other hand, due to the economy's dominant agricultural nature and struggles with unemployment and population outflow, Mecklenburg achieved a lower growth (4.8). This condition was primarily due to the attractiveness of living in the border regions on the German side compared to Poland and the sizable political intervention of German governments directed at these areas.

#### LAU Level

Synthetic component indicators (w1, w2) were analysed to illustrate the differentiation of material and non-material conditions of the population's life in the spatial aspect at LAU.

**Fig. 3:**
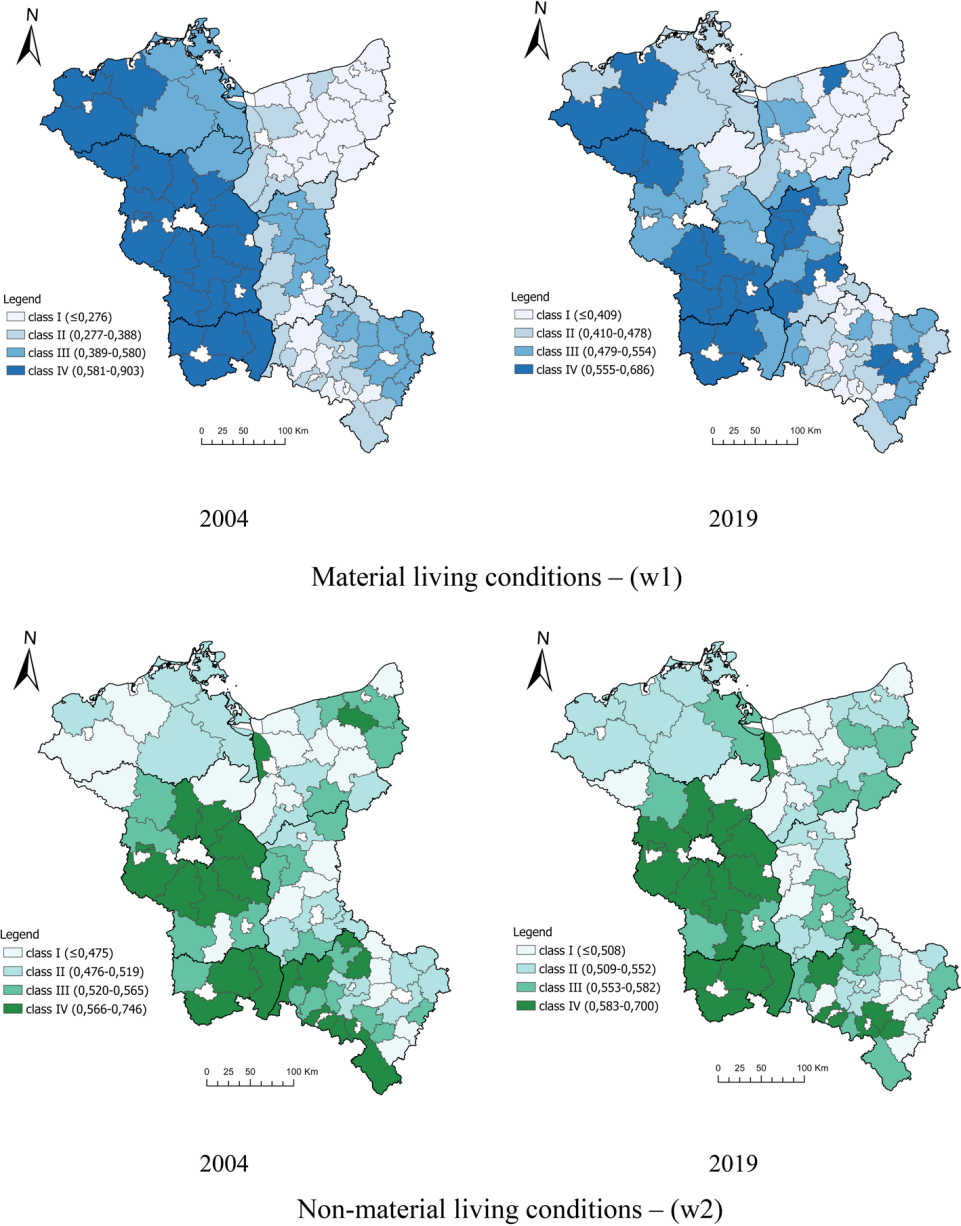
Spatial delimitation of the population's living conditions in the border regions of Poland and Germany at LAU in 2004 and 2019. Source: own calculations

The study results revealed significant differences in the population's material living conditions (w1) in the border regions in 2004 and 2019. The analysis showed a disproportionate distribution of spatial units within individual classes in 2004. The highest level of material conditions was located on the German side, while the lowest on the Polish side, concentrated mainly in Zachodniopomorskie (Fig. 3), where socialist agriculture played a significant role. In 2019 the differences in the material living conditions in the border regions of Poland and Germany were greater. However, ultimately the disparity between the material conditions in the border regions in Poland and in Germany decreased relatively (Fig. 3).

The level of non-material living conditions in border regions in 2004 and 2019 showed a relatively even distribution of units within individual classes. In 2004, on the German side, a visible concentration of units of classes II, III and IV was recorded. On the Polish side, the distribution of the analysed units was reasonably even. In 2019, there were no significant changes in the distribution of units in the German side's border regions. On the Polish side, more units qualified as classes II and III, which results from levelling non-material living conditions. Similarly, the results of spatial delimitation of non-material conditions in 2004 and 2019 in Poland and Germany's border regions were not significantly diverse and remained at a similar level (Fig. 3). This state of affairs may mean that, despite the measures introduced to improve the population's non-material conditions, more time is needed to notice them in spatial changes than in the case of material conditions.

**Fig. 4:**
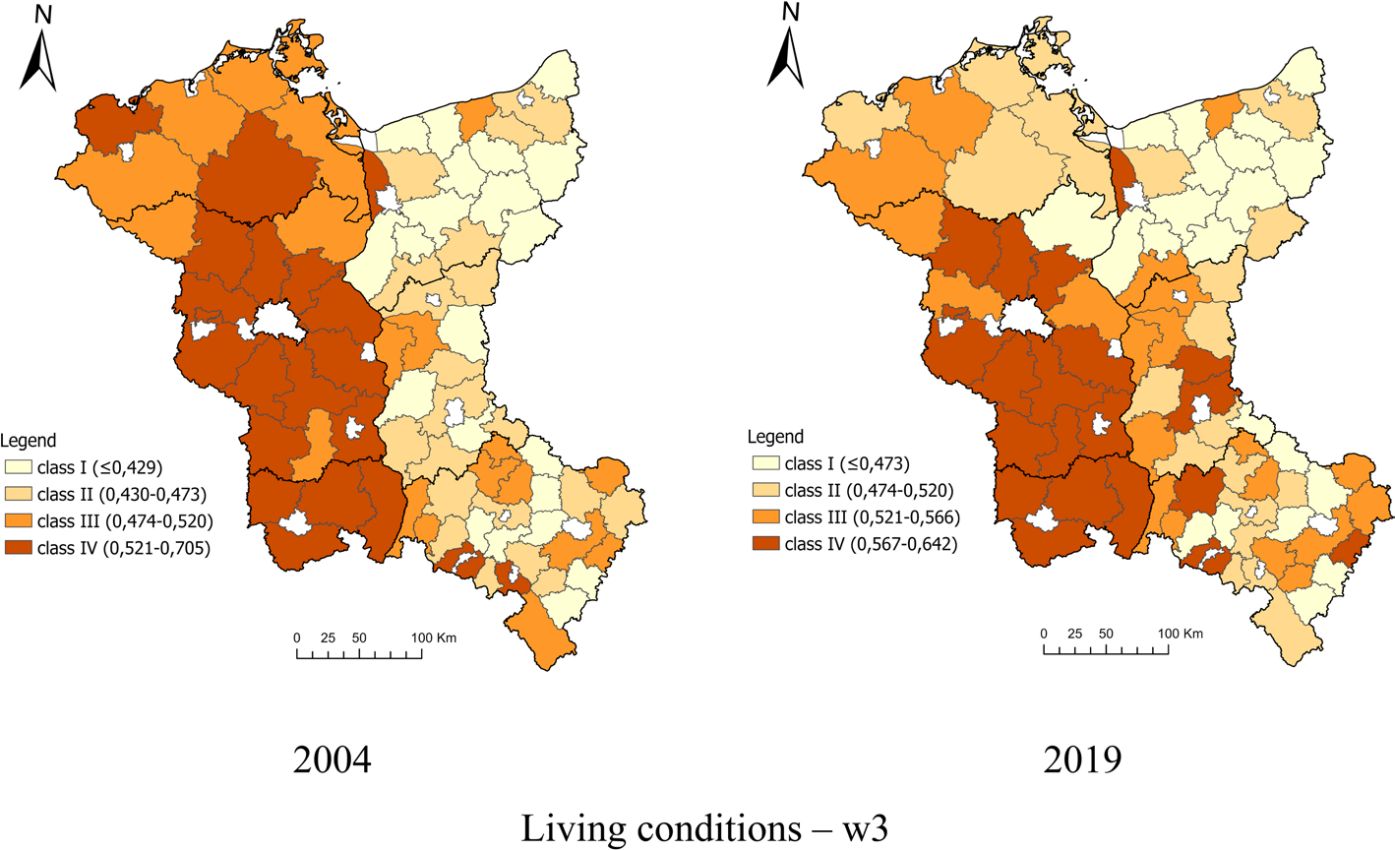
Spatial delimitation of the living conditions in the border regions of Poland and Germany at LAU in 2004 and 2019. Source: own calculations

The study of the general synthetic indicator of life quality (w3) revealed significant differences in Poland and Germany's border regions. In 2004, it was higher in German regions than in the Polish ones. On the other hand, in 2019, the distribution of units within individual classes on the German and Polish sides showed less differentiation, indicating a reduction in disproportions. The level of the synthetic indicator of life quality is noticeably high in densely populated areas with a better settlement network while it is the lowest in less populated areas. The study results clearly show that the living conditions both on the German and Polish side of the border deteriorate from north to south (w3) (Fig. 4). In 2004, there were significant spatial differences and significant disproportions between the border regions in Germany and Poland. In the context of territorial cohesion policy, this situation was unfavourable and indicated the competitiveness between these regions. On the other hand, the spatial delimitation of the living conditions in 2019 showed slow changes towards their levelling, influenced by the EU financial policy.

### Spatial policy of Territorial Cohesion—European Territorial Cooperation

The cross-border cooperation programmes along internal EU borders were funded by the European Regional Development Fund (ERDF) under INTERREG and from 2007—the European Territorial Cooperation (ETC) programme (Jakubowski et al., 2017). According to the “European Charter…, 2011”, cross-border cooperation should be understood as any joint activity aiming to strengthen and foster the development of neighbourly relations between communities and territorial authorities (European Commission, 2018).

European Territorial Cooperation (ETC), also known as INTERREG, is a relatively recent political process (Wassemberg et al., 2015) as it only became one of the main objectives of EU cohesion policy in 2007. It followed the successful implementation of the INTERREG Community Initiative (1990–2006). Cross-border cooperation programs were initially intended to help prepare border areas for the EU Single Market, promote administrative cooperation and reduce their isolation on the EU territory (EC 1990a, 1990b). These programs gradually turned into socio-economic support instruments for internal and external border areas of the EU (Medeiros, 2018). It is recognised as an additional policy instrument, complementing other EU regional development and cohesion policies to promote EU border regional development. Since 1990, five generations of INTERREG programs have been implemented (Table 5). INTERREG I (1990–1993), INTERREG II (1994–1999), INTERREG III (2000–2006), INTERREG IV (2007–2013) i INTERREG V (2014–2020) (Dołzbłasz, 2017) (Table 5). From INTERREG III, the program has been split into three separate components: (A) for cross-border projects, (B) for transnational projects and (C) for interregional cooperation, (Reitel et al., 2015, 2018).

**Table 5:** Evolution of INTERREG programs in 1990–2020. *Source*: own study on based on Dołzbłasz (2017)

Specification	INTERREG I 1990–1993	INTERREG II 1994–1999	INTERREG III 2000–2006	INTERREG IV 2007–2013	INTERREG V 2014–2020
Legal status	Community initiative		Included in the Structural Funds regulations		Own regulations
Number of Member States supported (EU internal borders)	11	At the beginning 11 countries at the end 15	In the beginning 15 countries in the end 25 countries	In the beginning 27 countries in the end 28 countries	28
Financial commitment (ECU)	1.1 mld	3.8 mld	5.8 mld	8.7 mld	10.1 mld

The main objectives of the INTERREG A programs were varied and included:

I. 1989–1993 Preparation of border areas for the Single Market, bearing in mind economic and social cohesion.
II. 1994–1999 Development of cross-border social and economic centres through common development strategies.
III. 2000–2006 Development of cross-border economic and social centres through joint strategies for balanced territorial development
IV. 2007–2013 Reducing the adverse effects of borders, such as administrative, legal and physical barriers; solving common problems and using untapped potential. Through joint management of programs and projects, mutual trust and understanding as well as the cooperation process are strengthened.
V. 2014–2020 Addressing mutual challenges identified jointly in border regions and exploiting untapped growth potential in border areas while enhancing the cooperation process for the overall harmonious development of the EU (Medeiros, 2018)

Implementation of EU’s cross-border cooperation programs has provided support for the creation of business partnerships between small and medium-sized enterprises (SMEs) and research centres, improvement of the physical accessibility of borders (Medeiros, 2014) and development of cross-border entrepreneurship (Smallbone & Welter, 2012). These programs supported political and partnership cooperation between small and medium-sized towns and cities while bringing together several authorities at different levels to deal with all kinds of border problems (Medeiros, 2018). The programs include: (I) promoting knowledge and experiences exchange; (II) developing strategic planning capabilities; (III) minimising negative externalities; (IV) improving joint management of natural resources; (V) improving access to transport and communication networks; (VI) developing joint use of infrastructure; (VII) supporting links between urban and rural areas; (VIII) promoting administrative capacity, employment and equal opportunities; (IX) developing multilingualism; (X) supporting research and innovation; (XI) aiding professional mobility and (XII) supporting spatial planning (Territorial Agenda, 2007) Each EU cross-border cooperation program was different, as each was tailored to cross-border region's specificities. Initially, cooperation was seen as an element to help overcome most border regions' peripherality characteristics (Blatter & Clement, 2000). With time, however, it was also treated differently, e.g. as cooperation to use the potential of border areas (border areas as a whole), or create cross-border regions, where an important role is played by, e.g. economic links (Blatter & Clement, 2000; Ciok, 2004; Dołzbłasz, 2017; Dörry & Decoville, 2012; Durand & Nelles, 2012; Gruchman et al., 2002; Knotter, 2014; Lara-Valencia, 2011; Spierings & Van der Velde, 2008). It was also pointed out that one of the cross-border areas' potentials might be their high tourist attractiveness and significant natural values. To determine the impact of the EU cohesion policy and the use of EU programs and funds on the population's living conditions, the following funding periods were adopted for the study: 2000–2006, 2007–2013, 2014–2020. The research covered INTERREG III, IV and V. In total, 27 programs implemented in the study area were analysed (Table 6). These were INTERREG cross-border (A) and interregional (C) programs. The research explored the Keep.eu website, which has extensive data on projects, programs, and partners of Territorial Cooperation with the European Union's participation since 2000.

**Table 6:** Analysed EU cross-border programs (INTERREG)

Nr	Programme	Programme area for the study area*
*Period 2000–2006*		
1	2000–2006 Brandenburg–Lubuskie (DE-PL)	B, L
2	2000–2006 Czech Republic–Poland (CZ-PL)	Do
3	2000–2006 ESPON I	B
4	2000–2006 Interact I	B, Dr, L, Z
5	2000–2006 Interreg IIIC East	B, Dr, Do, L, Z, MV
6	2000–2006 Interreg IIIC North	B, Dr, MV, Z
7	2000–2006 URBACT I	MV
8	2000–2006 Pamina (FR-DE)	B
9	2000–2006 Poland–Ukraine–Belarus (PL-UA-BY)	Do
10	2000–2006 Saxony–Czech Republic (DE-CZ)	Dr
11	2000–2006 Saxony–Poland (DE-PL)	Dr
12	2000–2006 Mecklenburg Vorpommern–Poland (DE-PL)	B, Dr, MV
*Period 2007–2013*		
1	2007–2013 Bavaria–Czech Republic (DE-CZ)	Dr
2	2007–2013 Czech Republic–Poland (CZ-PL)	Do
3	2007–2013 Interreg IVC	B, Do, Dr
4	2007–2013 Lubuskie–Brandenburg (PL-DE)	B, Do, L
5	2007–2013 Mecklenburg-Vorpommern/Brandenburg–Zachodniopomorskie (DE-PL)	B, MV, Z
6	2007–2013 Saxony–Czech Republic (DE-CZ)	Dr
7	2007–2013 Saxony–Poland (DE-PL)	B, Dr, Do, L, MV
8	2007–2013 South Baltic (PL-SE-DK-LT-DE)	MV, Z
*Period 2014–2020*		
1	2014–2020 Interreg Europe	B
2	2014–2020 INTERREG V-A Czech Republic–Poland	Do
3	2014–2020 INTERREG V-A Germany/Brandenburg–Poland	B, L
4	2014–2020 INTERREG V-A Germany/Mecklenburg–Western Pomerania / Brandenburg – Poland	B, MV, Z
5	2014–2020 INTERREG V-A Germany/Saxony–Czech Republic	Dr
6	2014–2020 INTERREG V-A Poland–Denmark–Germany–Lithuania–Sweden (South Baltic)	MV, Z
7	2014–2020 INTERREG V-A Poland–Germany/Saxony	Do, Dr, L

^*^B—Brandenburg (de); Dr—Dresden (de); MV-Mecklenburg-Vorpommern (de); L—Lubuskie (pl); Do—Dolnośląskie (pl); Z- Zachodniopomorskie (pl)

Table 6 shows the relationship between the programs and the support area at NUTS 2 units in the analysed region. Programs were grouped into three periods. Most of them were carried out in Brandenburg (as many as 14), and the least in Lubuskie and Zachodniopomorskie (7 each).

**Fig. 5:**
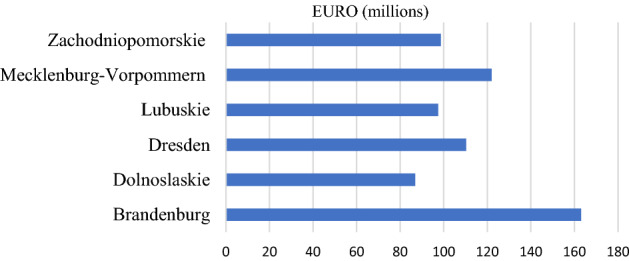
Absorption of EU funds (INTERREG) by NUTS 2. Source: own calculations

**Fig. 6:**
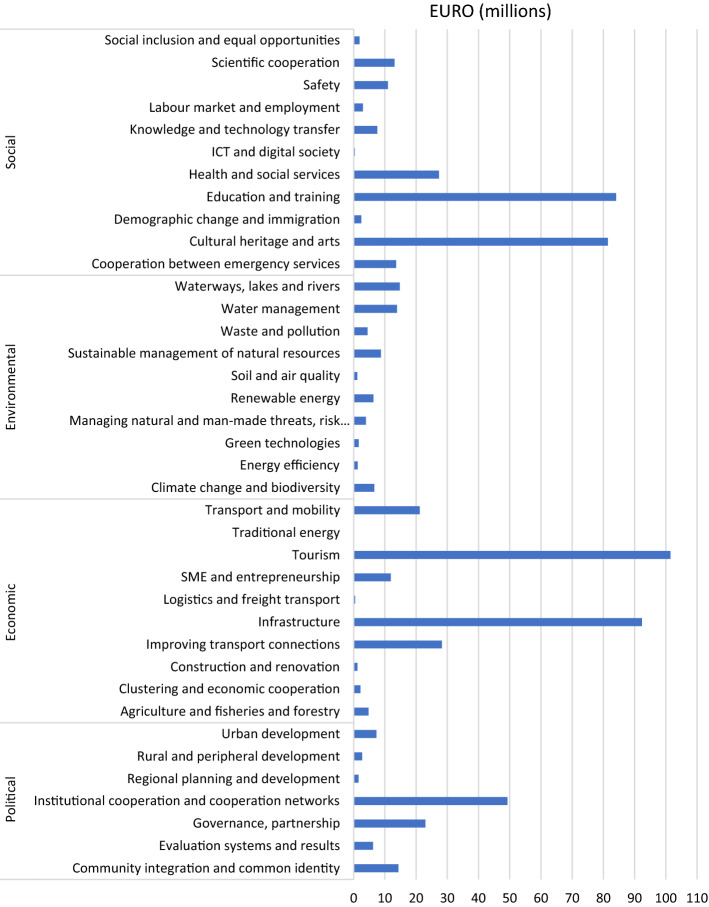
Absorption of EU funds (INTERREG) by the thematic scope of activities. Source: own calculations based on Keep.eu

**Fig. 7:**
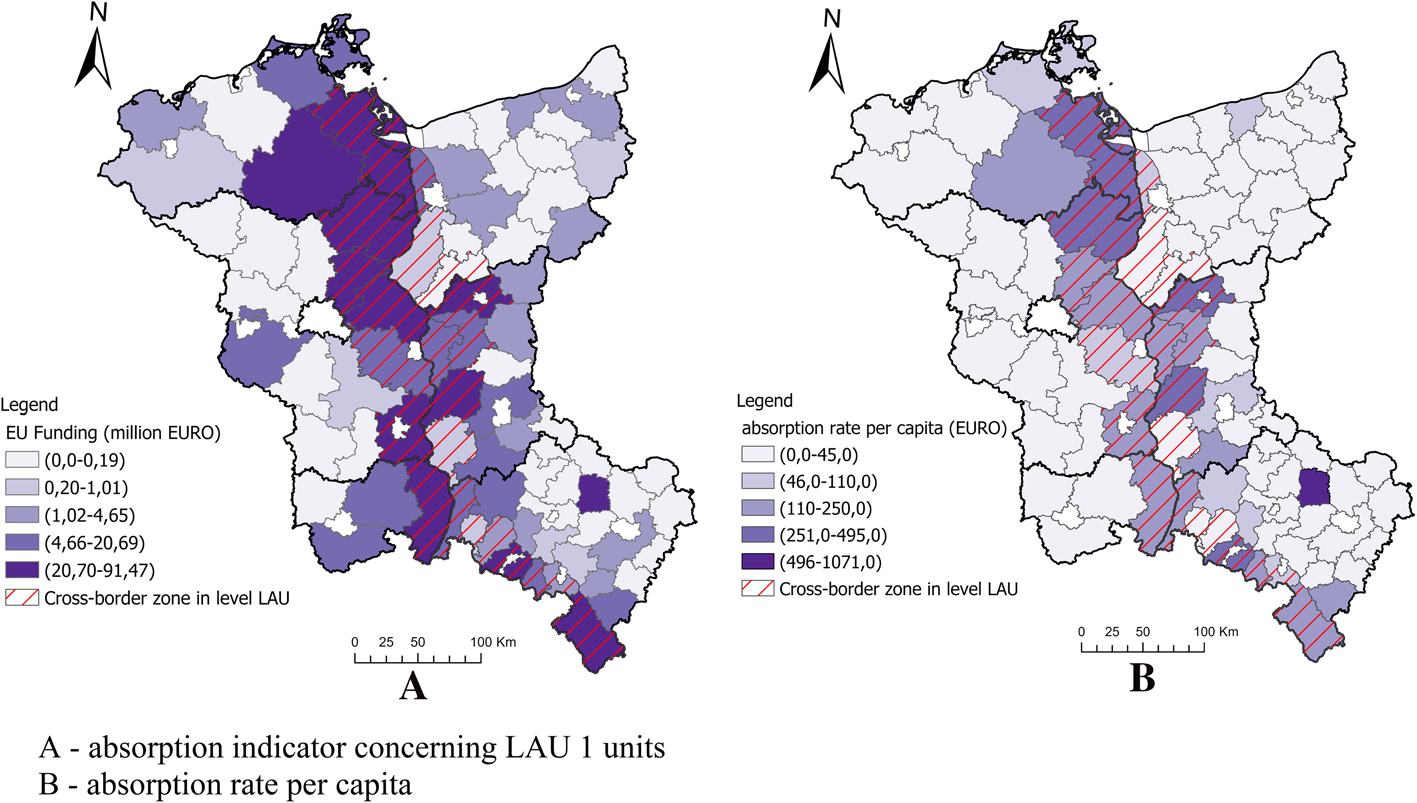
Financial resources obtained from EU programs and funds in the border regions of Poland and Germany at LAU in 2000–2020. Source: own calculations

The obtained support from the EU funds (INTERREG) for the analysed spatial units at NUTS 2 is presented in Fig. 5. Since 2000, the highest support of over EUR 160 million was obtained by Brandenburg, while the lowest of over EUR 86 million by Dolnośląskie. It should be emphasised that Poland became a member of the EU in 2004, which resulted in the fact that it used the INTERREG III programs of 2000–2006 to a small extent. Therefore, the level of absorption of EU funds is lower for voivodeships in Poland. In order to illustrate the directions of support, the thematic structure of the projects was analysed. The starting point was to define the thematic scope of joint actions based on the categories of the intervention of EU structural funds (Theme 1). The thematic scope of activities was grouped into economic, social, social and political factors (Fig. 6). The highest support was directed to activities in the social and economic categories. Most EU funds were spent on activities in tourism and infrastructure, Education and training and Cultural heritage and arts. These activities were intended to ensure equal chances, that is, territorial cohesion, in access to the employment market, health care and education. Funds transferred from INTERREG to the Polish border region were mostly allocated to combating poverty and social exclusion which for many years have been on the political agenda of the European Union (Europe 2020 Strategy). Jakubowski et al. (2017) presented similar results. They pointed out that in 2007–2013 most cross-border cooperation projects involving Poland implemented along EU internal borders were associated with supporting the development of tourism, transport infrastructure, relations and cooperation, as well as cultural projects (Jakubowski et al., 2017). The amount of support for INTERREG programs for LAU areas was presented. The highest support was granted to the areas located directly at the border (Fig. 7), mainly targeted at cross-border support. The adopted indicator relative to per capita confirms that the INTERREG support is directed mainly to the areas located at the border, and the higher level of support was granted to the areas on the German side (Fig. 7).

### Identification of the Relationship Between the Living Conditions of the Population and the Impact of EU Funds as an Essential Instrument of Spatial Policy

The research results show that the activities undertaken within the framework of cross-border cooperation in the spatial territorial cohesion policy were primarily aimed at improving the population's living conditions by compensating and supplementing them rather than exacerbating the differences resulting from competitiveness. It is evidenced by the lack of correlation between the synthetic indicator for assessing the living conditions of the border regions' population (2004 and 2019) and the absorption indicator (2004–2019). The high level of living conditions did not generate a high level of EU funds absorption from INTERREG programs. Most of the measures were targeted at the areas with a low level of living conditions (Fig. 4). The spatial disproportions in the population's living conditions noted in 2004 in Poland and Germany's border regions were alleviated by 2019. The INTERREG programs for units located in the state border's immediate vicinity played a special role in this respect, which is often associated with certain restrictions related to economic activity and resulting from their location. Therefore, to fully detail the research on identifying the relationship between the population's living conditions and the impact of EU funds, a correlation was made between LAU units located near the state border and their indicator of living standards. The research covered 22 rural units located in the German (7) and Polish (15) border zones. Based on the data showing the dynamics of the living conditions level, it was noticed that Polish units with improved living conditions at the same time obtained higher financing from INTERREG funds. The research results show that the spatial policy of territorial cohesion of the EU and the cross-border programs (INTERREG) contributed to the increase in the border area population's living conditions (Table 7). The effect of cross-border cooperation is more equal level of living conditions on both sides of the border in LAU units in 2019 compared to 2004.

**Table 7:** Classification of the living conditions of the population and funds from the European Union INTERREG 2004 program at border LAU. *Source*: own calculations

Specification	Cross-border at level LAU					
	Total		In the German border zones		In the Polish border zone	
*Living conditions*						
Class	2004	2019	2004	2019	2004	2019
Unfavourable	8	6	5	4	3	2
Medium low	7	8	2	1	5	7
Medium high	5	5	0	1	5	4
High	2	3	0	1	2	2
Total	22	22	7	7	15	15
*Change in the level of living conditions*						
Class	2004		2019		2004	
Improvement	6		3		3	
No change	12		4		8	
Degradation	4		0		4	
Total	22		7		15	
*Financial resources obtained from EU programs and funds from INTERREG in 2000–2020*						
Very low	4		0		4	
Low	1		0		1	
Medium	4		0		4	
Hight	10		4		6	
Quite high	3		3		0	
Total	22		7		15	

### Policy Implications—Competitiveness or Complementation?

Due to their peripheral location, border zones are areas of both competition and complementation. These relations taking place in the process of European integration may be subject to solid intensification. The EU cohesion policy's financial instruments can help break down barriers and facilitate the establishment and implementation of cross-border cooperation at various levels, including the living conditions. Financial instruments of the EU cohesion policy can help in breaking down barriers and facilitating the establishment and implementation of cross-border cooperation at various levels, including the living conditions of the population. Simultaneously, they may increase the competitive pressures from the areas located on the other side of the border.

Based on the above research on the living conditions of the population, some policy implications are presented.

Firstly, in terms of material conditions, the labour market, i.e. the unemployment rate, which was much lower than in the German border zone, was of most importance in shaping the Polish zone's competitive position. Regarding non-material conditions, the most critical aspect was education, i.e. the number of primary schools per 100 km^2^, much higher on the Polish side; on the German side it was healthcare, i.e. the number of people per 1 hospital bed. Bearing in mind better access to medical care in Germany, regional self-governments should establish cooperation in this area and facilitate Polish citizens' access to German hospitals and medical facilities. Similarly, better accessibility to schools in Poland can create international schools with both Polish and German students. An example of such a school is Europaschule Marie & Pierre Curie w Guben.

Secondly, the attractiveness of the place had a vital role in shaping competitiveness. In the German border zone, water and sewage infrastructure played a more significant role, i.e. the share of people using sewage treatment plants as % of the total population, while in the Polish zone, the share of forests in the total area. The natural value could be used to develop tourism and recreation for the inhabitants on both sides of the border.

Thirdly, in the border area, changes in the importance of factors influencing the region's competitiveness regarding the population's living conditions have been noted. In the Polish zone, the factors determining the labour market were the dominant ones. The German zone's dominant factor was demography (an increase in the migration balance per 1000 population). Therefore, local governments of LAU 1 units should take further actions towards cross-border cooperation within the territorial cohesion policy. Fourthly, the observed activities are consistent with the challenges identified by the European Commission that in the New Strategic Agenda for 2019–2024 claimed combating inequality and social exclusion to be the most important challenges. The internal border of the EU is not a barrier to development but can create an opportunity to establish cooperation in various areas of social and economic life, depending on the needs. The result is socio-economic relations the benefits of which are seen on both sides of the border. The coordination of funds at different socio-economic levels must be continued to ensure that the joint cross-border activities are spatially complementary. The results comply with the results of studies on the border of Romania (Mitrică et al., 2017) and Bulgaria (Kulcsár, 2014), Hungary, Serbia and Ukraine (Boar et al., 2010).

Fifthly, the situation is different in areas within the external EU border region. The studies evaluating territorial, social and economic cohesion under EUBORDERREGIONS (2015) showed that these are mostly border projects and not cross-border projects that increase the competitiveness of regions. Therefore, joint undertakings on both sides of the border should be reinforced. The main obstacles are the administrative and legal system and the Schengen Area visa policy (Kozak & Muça, 2020).

## Conclusion

The research showed that the border regions' level of living conditions was spatially diversified. It should be emphasised that border regions of both Poland and Germany showed less favourable diagnostic indicators describing living conditions in the material and non-material aspects than the country's corresponding average value. Thus, the research confirmed the existing relationship between border regions and central areas raised in the core and periphery theory. The results are in line with the evidence presented to the Council and the European Parliament (COM (2017) 534 final) which also noted that the EU's internal border regions perform worse than other regions in the home Member State. Simultaneously, to prevent the accumulation of negative processes in the border regions, various activities are carried out to stop them and implement cooperation between the regions on both sides of the border, which positively affects their socio-economic development. The research proved that the territorial cohesion policy and its cross-border programs (INTERREG) played an essential role in eliminating the differences in living conditions, which confirmed their importance primarily for units located in the border area. Positive results referred to building relations, common identity, and promoting openness and tolerance towards other nations. The programmes also widely contributed to decreasing barriers to cooperation and improving social integration (Binder & Matern, 2020).

Moreover, significant changes in border regions' living conditions in 2004–2019 were established. In 2004, the border between the regions within LAU units located on the German and Polish side was visible in the spatial context. The earlier membership of East Germany in the EU and the possibility of using funds and programs prepared for the member states were not without significance for this fact. Poland's accession to the European Union also made it possible to use the programs and at the same time facilitated the establishment of cross-border cooperation between regions on both sides of the border of Germany and Poland, which became an internal border of the EU. The effects of these activities are visible for individual units at LAU in 2019. The spatial delimitation of living conditions showed slow changes in shaping Poland's living conditions and Germany's border regions towards their levelling, influenced by the spatial cohesion policy. Disparities in material living conditions were less clear on both sides of the border than disparities in non-material conditions. This state may mean that in order to notice a significant improvement in the non-material conditions of the population in shaping space, more time is needed than in the case of material conditions, where first there are quantitative and then qualitative changes. It means that further activities, especially in the border area constituting a shared service and commercial base for residents on both sides of the border, should focus on improving the availability of social and technical infrastructure and space for recreation, including tourism development. Other activities in this area would shape a territorial cohesion policy based on competitiveness but aiming at complementation.

Research on the population's living conditions using social indicators, enabling comparing different regions, is needed to establish guidelines for shaping spatial policy and area development practices. By comparing cross-border areas within the EU, we can provide more informed and substantive discussions on improving living conditions on both sides of the border. Further assessment, especially long-term, is needed to understand changes over time, including those in policies and living conditions. This research is very much needed, especially in border regions, given the current geopolitical climate in the context of the Covid 19 pandemic (temporary border closure), which is particularly difficult in areas that aim to achieve territorial cohesion precisely through complementation. There is a need to develop procedures that would allow the implementation of the spatial policy pursued despite its restrictions.

A comparative analysis of the living conditions in the Polish and German border regions clearly implies a need for continuing cross-border cooperation, especially through social innovation. Border regions should in particular prevent inequality in living conditions through the exchange of knowledge, concentration of resources and satisfying local needs by undertaking joint activity and complementary projects. This applies to local stakeholders both from the market and public sector and citizens cooperating in order to satisfy local needs using knowledge.

### Electronic supplementary material

Below is the link to the electronic supplementary material.

Supplementary file1 (XLSX 34 kb)

## Appendix

See Table

**Table 8:** Descriptive statistics for the variables used in the analysis (LAU area), by year of observation

Year	2004				2019			
Variables	Min.	Max.	Mean	Std. dev.	Min.	Max.	Mean	Std. dev.
Registered unemployment rate	12.90	42.70	26.82	7.28	1.30	19.00	8.67	3.67
Vehicles per 1000 inhabitants	309.34*	559.20*	456.73	58.65	495.47	780.93	632.31	62.79
Number of rooms per person	1.12	3.99	1.56	0.61	1.29	2.39	1.66	0.35
Population per 1 hospital bed	77.54	963.01	325.24	210.19	83.57*	3533.43*	386.81	530.63
Number of primary schools per 100 km^2^	0.89	8.75	2.64	1.41	0.94	6.75	2.52	1.22
Population using sewage treatment plants in % of the total population	24.40	94.48	67.13	15.90	45.80	93.60	77.75	10.92
The share of forests in the total area (%)	6.23	59.68	33.04	12.70	6.49	60.16	33.70	12.73
Fatalities in car accidents per 100 thousand population	1.54**	36.97**	11.24	6.70	2.27	25.94	8.51	4.08
Net migration per 1000 population	− 11.04	18.90	− 1.03	5.53	− 7.33	28.12	1.25	5.96

*2009 **2011*2017 year

8
